# The Double-Edged Sword of Digital Engagement—How Digital Access and Internet Use Reshape Sleep Schedules and Underlying Mechanisms in Older Adults: Longitudinal Observational Study

**DOI:** 10.2196/79731

**Published:** 2025-11-05

**Authors:** Chi Zhang, Longxuan Lin, Li Wang, Han Hu, Heyang Zhang

**Affiliations:** 1 Lanzhou University, No. 222, Tianshui South Road, Chengguan District, Lanzhou, Gansu Province, 730000, China, 86 18759217889; 2 School of Philosophy and Sociology, Northwest Normal University, Lanzhou, China; 3 School of Public Policy and Administration, Xi’an Jiaotong University, Xi'an, China

**Keywords:** digital health, digital engagement, sleep duration, afternoon nap, sleep onset time, mediation effect, cohort study

## Abstract

**Background:**

Given the rapid development of the digital economy and the sustained proliferation of the internet, digital engagement in older adults has garnered mounting attention from the academic community. However, research has yet to systematically examine the impact of digital engagement on sleep in this demographic.

**Objective:**

This study aims to examine the association of digital engagement—operationalized as digital access and internet use duration—with the sleep schedules (nocturnal sleep duration, afternoon nap duration, and sleep onset time) of older adults in China, using longitudinal data and robust statistical modeling to explore longitudinal associations and potential mechanisms.

**Methods:**

Data were derived from 4 waves (2014, 2016, 2018, and 2020) of the China Family Panel Studies, involving 16,784 older adults (≥60 y). We used panel fixed effects models and a random-effects ordered logit model to analyze the effects on continuous outcomes (nocturnal and nap sleep duration), controlling for time-invariant individual characteristics. As sleep onset time is an ordinal variable, a random-effects ordered logit model was used for this outcome. Moderation analyses were conducted by introducing interaction terms (digital engagement×sex and digital engagement×residence) into the models to examine heterogeneity across subgroups (urban or rural, men or women). Mediation analyses were performed using the Sobel test with year-fixed effects and the nonparametric bootstrap method (1000 resamples) to assess the significance of indirect effects via mechanistic pathways (nonfarm employment, protein intake, memory, depressive mood, and instrumental activities of daily living).

**Results:**

The study included a total of 16,784 older adults, with an average age of 69 (SE 6.946) years, including 9100 (54.22%) women and 7684 (45.78%) men. The results showed that both digital access (β=−.15, 95% CI −.25 to −.06; *P*=.002) and internet use time (β=−.07, 95% CI −.13 to −.01; *P*=.027) were significantly associated with significantly shorter sleep duration of older adults. Digital access was significantly associated with a significant reduction in the length of afternoon naps among older adults, while internet use did not have this effect; both digital access and internet use were significantly associated with a significant delay in older adults’ sleep onset time. Digital access was associated with older adults’ sleep schedules through its correlations with nonfarm employment, protein intake, memory, depressive mood, and instrumental activities of daily living. Digital access had a greater and more significant impact on men and urban older adults, while internet use had a greater and more significant impact on women and urban older adults.

**Conclusions:**

The study indicates that digital engagement, such as the use of electronic devices, is associated with a reduction in both daily and nap sleep duration, as well as a delay in sleep onset, among older adults.

## Introduction

The digital engagement of older adults has become a subject of increasing interest and concern within the academic community. This phenomenon is precipitated by the rapid development of the digital economy and the continuous popularization of the internet [1-3]. In Europe and the United States, the proportion of individuals aged ≥65 years who use the internet has increased from less than 30% (the United States in 2004 and Europe in 2010) [4,5] in the early years to approximately 60%‐67% in recent years (the United States in 2016 and Europe in 2019) [5,6]. In a similar vein, forecasts suggest that the internet penetration rate among individuals aged ≥60 years in China will reach 51.07% in 2023 [7,8]. Despite the numerous benefits of internet use for older adults [9-11], concerns have been raised regarding its impact on sleep. Specifically, internet use has been shown to potentially disrupt the duration and quality of sleep by occupying sleep time [12], interfering with physiological rhythms [13], increasing brain excitation [14], and interfering with the sleep environment, among other factors. The impact of digital engagement on the sleep of older adults warrants further in-depth study.

Previous studies have shown that sleep is influenced by multiple factors including physiology [15,16], psychology [17,18], environment [19-21], and behavior [22-24], but this study focuses on the digital engagement factors among them. The digital engagement is significantly associated with 4 primary factors that influence sleep in older individuals: the use of LED-backlit screens on electronic devices has been demonstrated to emit high-intensity, short-wavelength blue light, which has been shown to inhibit melatonin secretion [25,26]. A growing body of research has indicated that online social media use can reduce feelings of loneliness and depression in older adults [27-29]. In addition, digital engagement has been found to disrupt sleep environments, such as the intrusion of electronic devices into bedrooms [30]. A sedentary lifestyle and lack of exercise due to internet access have also been shown to be detrimental to healthy sleep. The paucity of physical activity and sedentary lifestyle that is concomitant with Internet access has also been demonstrated to be deleterious to the quality of sleep in older adults [31]. However, there is a paucity of research on the impact of digital engagement on the sleep of older people. Digital delivery of cognitive behavioral therapies has been shown to have a positive effect on insomnia symptoms in older adults, with studies indicating a reduction in sleep effort [32,33]. In addition, community-based digital mental health monitoring platforms have the potential to provide real-time tracking of sleep fragmentation, efficiency, and other indicators, thereby assisting older adults in optimizing their sleep through a feedback mechanism [34]. However, it is important to note that nighttime use of digital screens by older adults may potentially exacerbate sleep disorders due to the suppression of melatonin by blue light [35]. A paucity of studies exists on the effects of digital engagement on older adults’ sleep and the mechanisms of its influence. Furthermore, these studies are unsystematic. However, given the increasing prevalence of digital engagement among older adults, it is imperative to investigate its association with sleep quality.

In summary, given the limitations of extant studies, this study endeavors to use the 4-period tracking data from the China Family Tracking Survey from 2014 to 2020. The study uses panel fixed effects (FE) models and a random-effects ordered logit model to investigate the impact of digital engagement (digital access and internet duration) on the sleep schedules of Chinese older adults aged ≥60 years. The dependent variables (sleep schedules) include sleep duration, afternoon nap duration, and nocturnal sleep onset time. The study also explores the underlying mechanisms by which these effects occur.

## Methods

### Design and Study Population

This study is based on data from the China Family Panel Studies (CFPS), a nationally representative, longitudinal survey launched in 2010 by the Institute of Social Science Survey at Peking University. The CFPS adopts a multistage probability proportional to size sampling strategy, covering 25 provinces, municipalities, and autonomous regions in China, which represent about 95% of the national population. The survey collects extensive information at the individual, household, and community levels, including socioeconomic status, health, education, family structure, and digital engagement. It is conducted biennially, thereby enabling longitudinal analysis of social, economic, and health transitions among Chinese residents. The CFPS is widely regarded as one of the most authoritative and comprehensive datasets for social science and public health research in China, and it has been extensively used in previous studies to examine issues related to aging, health, and digital behavior. The database under consideration includes respondents’ digital engagement and sleep schedules, which meet the needs of this study. The data used in this study to examine the impact of digital engagement on sleep schedules of older adults are drawn from 4 periods of CFPS in 2014, 2016, 2018, and 2020, comprising a total of 142,544 samples. This cohort study was conducted in accordance with the Strengthening the Reporting of Observational Studies in Epidemiology (STROBE) reporting guidelines (Checklist 1) [36].

### Ethical Considerations

The protocols followed in the CFPS were aligned with the principles of the Declaration of Helsinki [37]. This research was reviewed and approved by the Beijing University Biomedical Ethics Committee (IRB00001052-14010). All participants in the CFPS provided written informed consent. The participants received a physical examination report for their participation. The data were deidentified and treated for confidentiality by anonymization and coding.

### Sleep Schedule

The explained variable of this study is the sleep schedule of older adults, which is systematically operationalized as sleep duration, afternoon nap duration, and sleep onset time. The prevalence of regular napping among older adults has been well-documented [38-40]. However, the incorporation of this behavior into the broader context of sleep scheduling has been a notable oversight in previous research. The fixed-distance variables in hours include sleep duration and nap duration. Sleep onset time is an ordinal variable, combining the data at midnight to midnight the following day, and assigning the data from 1 AM to 4 AM to 5 AM to 8 AM for the sake of analyzing convenience. Concurrently, the atypical samples characterized by nocturnal sleep duration between 5 AM and 6 PM were eliminated from the analysis (out of 16,784 samples, 358 samples were thus excluded). With regard to the treatment of extant studies, sleep schedules were classified into 3 categories during the descriptive statistics stage [41].

### Digital Engagement

The core explanatory variables of this study are the digital engagement of older adults. Informed by prior research and the data structure of the CFPS, we operationalized digital engagement through two distinct but complementary measures that allowed us to distinguish between the binary state of being connected and the intensity of use among connected individuals:

1. Digital access: this is a binary variable indicating whether an older adult has fundamental access to the digital world. It was constructed based on responses to the CFPS questionnaire regarding the use of digital devices to access the internet. Respondents who reported using either a mobile phone or a computer to go online were assigned a value of 1. Those who used neither of these devices for internet access were assigned a value of 0.
2. Online time: this continuous variable measures the intensity of digital engagement among those with access. It is based on the self-reported average number of hours per week spent on the internet. To address the substantial right-skewness in its distribution (where most users report low-to-moderate hours, and a few report very high hours), we applied a natural logarithmic transformation to this variable. This transformation improves the linearity of its relationship with the sleep outcome variables and mitigates the influence of extreme values, thereby yielding more robust and interpretable regression coefficients.

It is important to note the analytical implication of this 2-pronged approach: the variable online time was only analyzed for the subsample of older adults who had digital access (ie, where digital access=1). For respondents without digital access, data on online time were logically defined as missing and excluded from analyses involving this specific variable.

### Covariates

The following covariates were used in this study: sex (assigned a value of 0 for females and 1 for males); marital status (assigned a value of 1 for those in marriage and 0 for those not in marriage); years of education; domicile (assigned a value of 0 for those in rural areas and 1 for those in urban areas); and age. The participants were asked to self-assess their health using a scale from 1 to 5, with 1=“very good health” and 5=“very unhealthy.” The higher the value assigned, the more unhealthy the participant’s health. The participants were also asked to indicate whether they had a religion (with the option of assigning a value of 1 for those who had a religion and 0 for those who did not) and chronic disease (presence of a chronic disease is assigned a value of 1, otherwise 0).

The selection of covariates was guided by their established associations with sleep patterns in the existing literature, aiming to minimize potential confounding bias. Sex, marital status, education, domicile (urban or rural), age, self-rated health, and chronic disease status are well-documented demographic and socioeconomic determinants of sleep. Although religious belief was reported by a small minority (542/16784, 3.23%) of our sample, it was retained as a covariate due to its theoretical relevance as a sociodemographic marker that can influence lifestyle, social support, and psychological well-being, all of which are linked to sleep quality and duration. We also considered including working status (eg, retired); however, this variable in the CFPS is primarily defined around formal urban retirement systems. Its inclusion would have drastically reduced the analyzable sample size and shifted the analysis to a nonrepresentative subpopulation, as the vast majority of respondents, particularly those in rural areas or with informal employment backgrounds, were not covered by this definition. Therefore, to maintain sample representativeness and statistical power, we opted not to include this variable.

### Statistical Analysis

Descriptive statistics were presented as means (with SDs) for continuous variables and counts (with percentages) for categorical variables. To estimate the longitudinal association of digital engagement with sleep schedules, we used panel regression models to control for unobserved time-invariant individual heterogeneity. For the continuous dependent variables (nocturnal sleep duration and afternoon nap duration), we estimated panel FE models. The FE model effectively controls for all time-invariant confounders by leveraging within-individual variation over time. This process inherently accounts for within-individual correlation by effectively comparing each person to themselves across different survey waves. Year-FE were included in all models to account for common temporal shocks affecting all respondents in a given survey year. For the ordinal dependent variable (sleep onset time), a random-effects ordered logit model was used. This model was chosen because the conditional FE estimator for ordered outcomes is subject to the incidental parameters problem in short panels, which leads to biased coefficient estimates [42]. The random-effects model accounts for within-individual correlation across survey waves by including an individual-specific random intercept, which follows a logistic distribution.

We conducted mediation analyses to test the hypothesized mechanisms through which digital access affects sleep schedules. We employed separate mediation models for each mediator to isolate its unique indirect effect. The significance of the mediation (indirect) effect was tested using two methods: (1) the Sobel test and (2) the nonparametric bootstrap method with 1000 resamples to generate bias-corrected 95% CIs. Mediation was considered statistically significant if the bootstrap CI did not include 0. All mediation models controlled for year-FE and the full set of covariates listed in the Covariates section.

To examine whether the effects of digital engagement differed across subpopulations, we performed heterogeneity analyses by introducing interaction terms between the digital engagement variables (digital access and internet use) and 2 key moderators: sex and urban-rural residence. Specifically, we added the interaction terms “digital engagement×sex and digital engagement×household” to our baseline models. Stratified models were then used to estimate and report the subgroup-specific coefficients.

Of note, the variable internet use duration was only applicable to and analyzed for the subsample of older adults who had digital access (ie, digital access=1). For those without digital access, data on internet use duration were treated as missing and excluded from the respective analyses.

## Results

### Sample Characteristics

**Figure 1.**
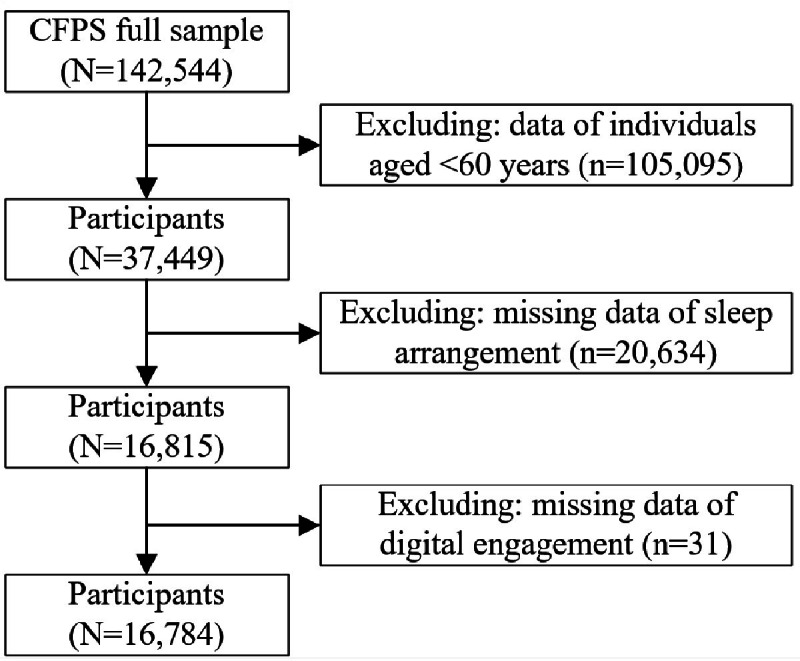
Flowchart of the study sample inclusion process. CFPS: China Family Panel Studies.

A total of 16,784 older adult samples were included in the study, including 9100 (54.22%) females and 7684 (45.78%) males with an average age of 69.726 (SD 6.95) years, after gradually excluding samples aged <60 years and samples missing digital engagement or sleep schedule (Figure 1).

Of the 2266 digital accesses and 15,418 digital divides, it is evident that there is considerable potential for further digital penetration among the older adult population in China. The digital access samples spent an average of 8.56 hours per week on the internet, suggesting a relatively balanced use pattern. Table 1 presents the fundamental characteristics of the samples according to their sleep schedules. The findings indicate that, in contrast to older adults experiencing digital exclusion, those who attained digital access demonstrated a reduced prevalence of extended or diminished sleep duration. Notably, the proportion of individuals experiencing protracted sleep duration was considerably lower in the study population compared to older adults within the digital divide. With regard to the duration of the afternoon nap, older adults with digital access predominantly demonstrate a tendency toward significantly abbreviated breaks, exhibiting a frequency approximately 13% higher than older adults encountering barriers to digital engagement. With regard to nocturnal somnolence, older adults with digital access exhibited a more balanced nocturnal sleep pattern, characterized by a reduction in very early sleep onset times and an increase in very late sleep onset times. In general, older adults who were male, had a high level of education, were married, had a rural household registration, and had no religion exhibited more balanced sleep duration. Similarly, older adults who were male, were married, had an urban household registration, and had no religion demonstrated more balanced afternoon nap duration. Finally, older adults who were female, had a high level of education, had an urban household registration, and had a religious affiliation exhibited healthier nocturnal sleep onset time.

**Table 1.** Baseline characteristics of participants according to sleep arrangement.

Characteristics	Total sample	Sleep per day (h)			Afternoon nap per day^a^ (h)			Sleep onset time^b^		
		≤5 (n=2283)	Approximately 5-9 (n=11,039)	≥9 (n=3462)	≤0.5 (n=2379)	Approximately 0.5-1.5 (n=5166)	≥1.5 (n=3258)	≤8 PM (n=3007)	Approximately 8 PM to 12 AM (n=12,783)	≥12 AM (n=623)
Digital access, n (%)										
Yes	2266 (13.50)	244 (10.77)	1843 (81.33)	179 (7.90)	510 (33.03)	802 (51.94)	232 (15.03)	123 (5.49)	1916 (85.54)	201 (8.97)
No	14,518 (86.50)	2039 (14.04)	9196 (63.34)	3283 (22.61)	1869 (20.19)	4364 (47.13)	3026 (32.68)	2884 (20.35)	10,867 (76.67)	422 (2.98)
Online time^c^, mean (SD)	2.147 (1.255)	2.165 (1.180)	2.174 (1.226)	1.805 (1.619)	2.083 (1.237)	2.152 (1.202)	2.159 (1.326)	2.012 (1.492)	2.082 (1.251)	2.711 (0.932)
Sex, n (%)										
Female	9100 (54.22)	1518 (16.68)	5679 (62.41)	1903 (20.91)	1433 (25.55)	2586 (46.11)	1589 (28.33)	1635 (18.39)	6970 (78.39)	287 (3.23)
Male	7684 (45.78)	765 (9.96)	5360 (69.76)	1559 (20.29)	946 (18.21)	2580 (49.66)	1669 (32.13)	1372 (18.24)	5813 (77.29)	336 (4.47)
At marriage^d^, n (%)										
Yes	12,608 (75.12)	1565 (12.41)	8673 (68.79)	2370 (18.80)	1804 (21.98)	4028 (49.09)	2374 (28.93)	2051 (16.62)	9801 (79.42)	488 (3.95)
No	4176 (24.88)	718 (17.19)	2366 (56.66)	1092 (26.15)	575 (22.14)	1138 (43.82)	884 (34.04)	956 (23.47)	2982 (73.21)	135 (3.31)
Education, mean (SD)	5.002 (4.829)	4.467 (4.656)	5.614 (4.868)	3.370 (4.375)	5.667 (5.044)	5.501 (4.951)	4.152 (4.521)	3.102 (4.162)	5.358 (4.866)	6.995 (4.653)
Household, n (%)										
Rural	9321 (55.63)	1268 (13.60)	5577 (59.83)	2476 (26.56)	1107 (18.69)	2692 (45.45)	2124 (35.86)	2089 (22.90)	6831 (74.89)	201 (2.20)
Urban	7433 (44.37)	1008 (13.56)	5444 (73.24)	981 (13.20)	1266 (26.02)	2467 (50.71)	1132 (23.27)	911 (12.54)	5930 (81.65)	422 (5.81)
Age (y), mean (SD)	69.726 (6.946)	70.655 (7.147)	69.060 (6.649)	71.234 (7.417)	69.350 (6.647)	69.745 (6.868)	70.922 (7.190)	72.437 (7.393)	69.182 (6.695)	67.066 (5.644)
Self-rated health^e^, mean (SD)	3.622 (1.184)	3.959 (1.108)	3.501 (1.159)	3.785 (1.247)	3.612 (1.138)	3.575 (1.169)	3.716 (1.229)	3.779 (1.229)	3.584 (1.169)	3.596 (1.207)
Religious belief, n (%)										
Yes	542 (3.23)	88 (16.24)	324 (59.78)	130 (23.99)	98 (26.42)	158 (42.59)	115 (31.00)	93 (17.42)	430 (80.52)	11 (2.06)
No	16,228 (96.77)	2193 (13.51)	10,709 (65.99)	3326 (20.50)	2278 (21.85)	5008 (48.03)	3140 (30.12)	2912 (18.35)	12,343 (77.79)	612 (3.86)
Chronic diseases^f^, mean (SD)	0.339 (0.473)	0.432 (0.495)	0.316 (0.465)	0.350 (0.477)	0.337 (0.473)	0.355 (0.478)	0.367 (0.482)	0.349 (0.477)	0.336 (0.472)	0.340 (0.474)

a Missing data for 5981 (35.64%) participants.

b Missing data for 371 (2.21%) participants. Time points were recorded as before 8 PM (≤8 PM), 8 PM to 12 AM, and after 12 AM (≥12).

c Online time was measured as the natural logarithm of self-reported weekly hours online. This variable was only applicable to and calculated for the subsample with digital access (n=2266).

d Marital status: “married” includes those currently in marriage; “not married” includes those separated, divorced, widowed, or never married.

e Self-rated health was assessed on a 5-point scale from 1=very good to 5=very poor. A higher score indicates poorer self-perceived health.

f Chronic disease is presented as the count and its corresponding percentage of the column total for “yes.”

### Condition for Sleep Schedules According to Digital Engagement

**Figure 2.**
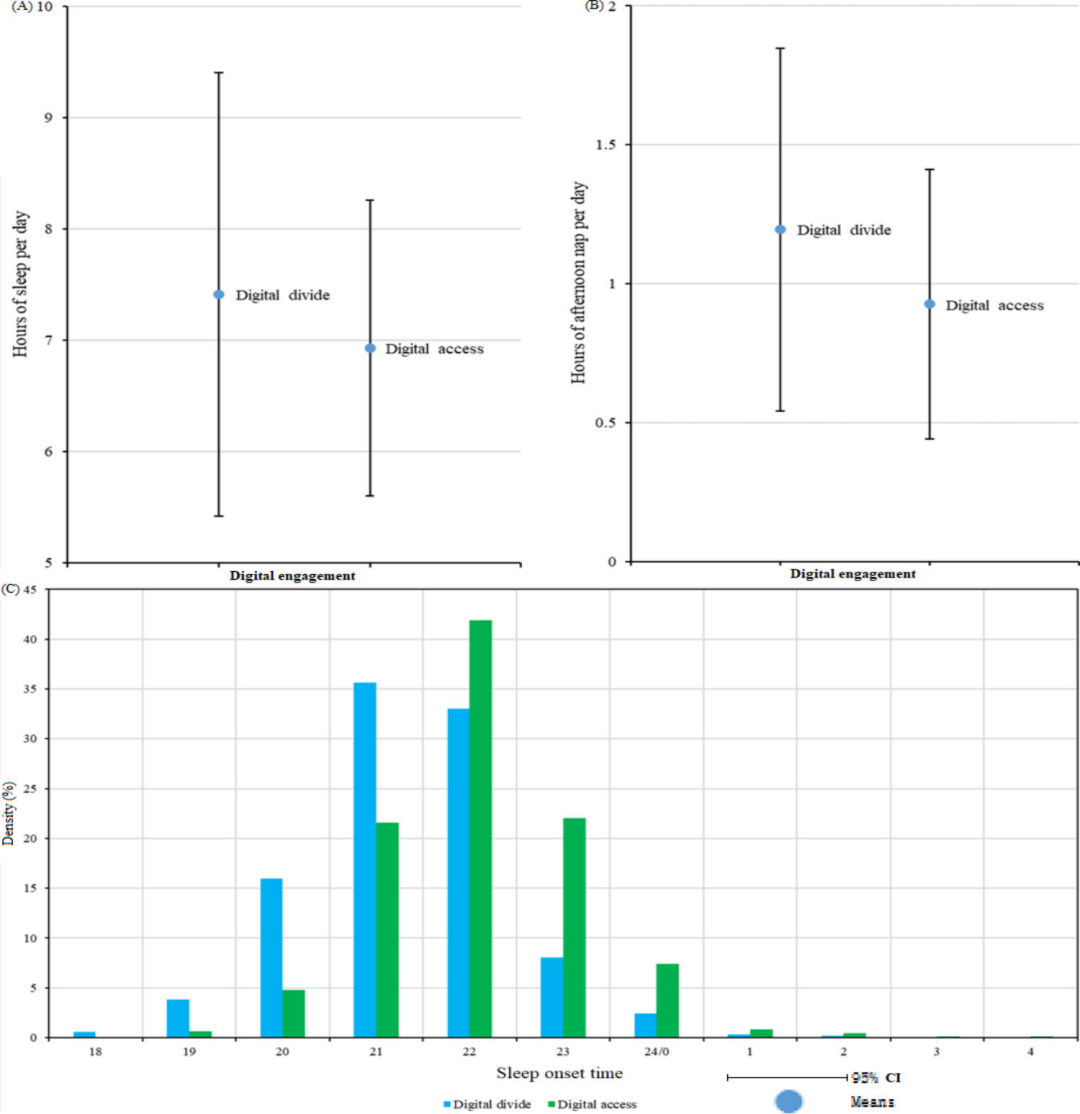
Condition for sleep schedules according to digital access. The density represents the probability distribution of each variable, allowing for visual comparison of the shape and central tendency of sleep schedules between older adults with (green) and without (blue) digital access. A higher density at a particular value indicates a greater proportion of the sample around that value. (A) Hours of sleep per day. (B) Hours of afternoon nap per day. (C) Sleep onset time.

**Figure 3.**
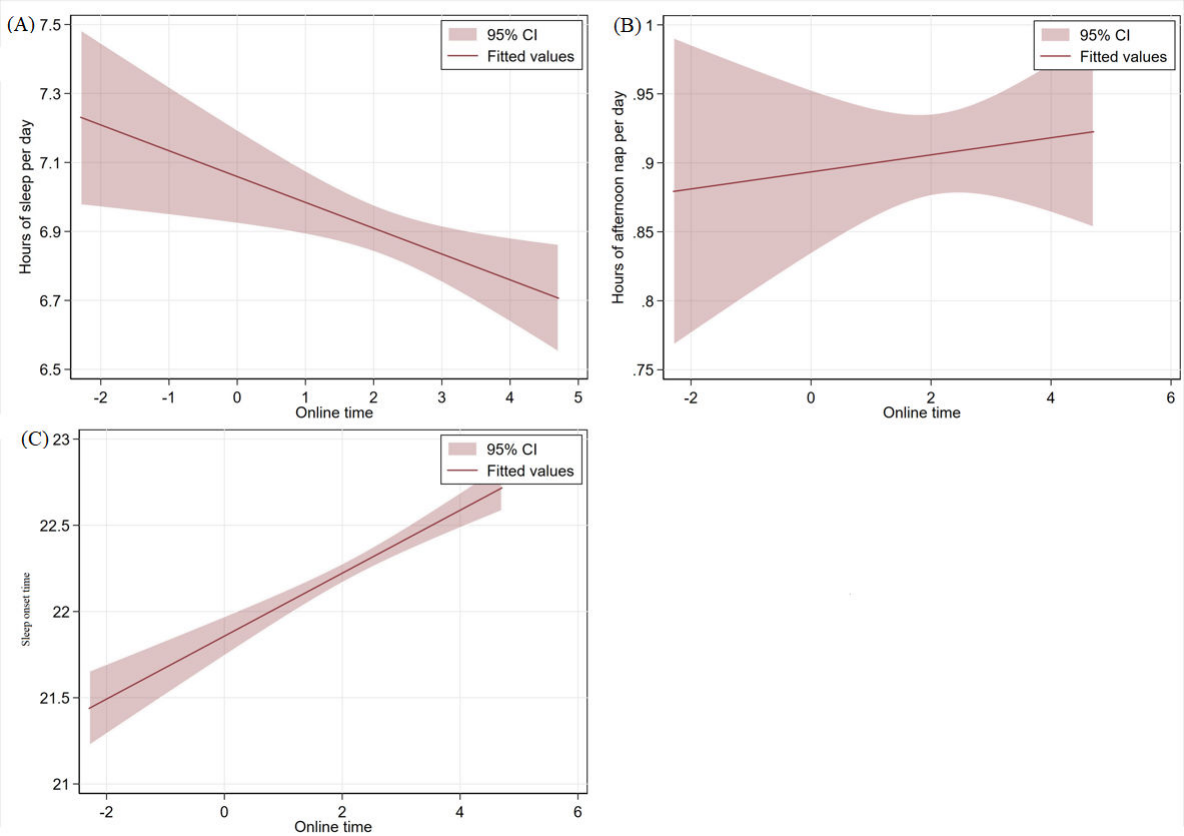
The correlation between online time and sleep schedules.

Figure 2 presents the sleep duration, afternoon nap duration, and nocturnal sleep onset time for older adults with varying digital access conditions. Figure 3 presents the correlation between online time and sleep schedules of older adults (nocturnal sleep onset time is an ordinal variable, but it can be approximated as a fixed-distance variable due to the large number of categories), with the sleep duration, afternoon nap duration, and nocturnal sleep onset time. The findings indicated that older adults experiencing the digital divide exhibited a greater number of daily sleep hours (mean 7.413, SD 1.990 h vs mean 6.929, SD 1.328 h) and more afternoon nap hours (mean 1.196, SD 0.653 h vs mean 0.926, SD 0.485 h) compared to older adults with digital access. With regard to the phenomenon of falling asleep, the overall tendency among older adults with limited digital access was to retire for the night at an earlier hour than their counterparts with digital access. The duration of time spent online exhibited a comparable tendency, with total sleep duration demonstrating an inverse relationship with the amount of time spent online. For older adults, the time of nocturnal sleep onset exhibited a positive correlation with the duration of time spent online. However, the length of the afternoon nap was proportional to the duration of internet access.

### Benchmark Regression

Table 2 reports the results of the baseline regressions (due to space limitations; Table S1 in the Multimedia Appendix 1), where model 1 is the effect of digital access on the length of daily sleep of older adults and model 2 examines the relationship between internet access duration and sleep duration among older adults. Model 3 explores the impact of digital access on the duration of the afternoon nap for this demographic. Model 4 analyzes the effect of internet access duration on the duration of afternoon nap for older adults. Model 5 investigates the impact of digital access on the timing of sleep onset for older adults during nighttime hours. Model 6 investigates the impact of internet access duration on the timing of sleep onset for older adults. The findings indicate that older adults with digital access were associated with a mean sleep duration of 0.154 hours less per day compared to those who may be experiencing a digital divide. In addition, a significant inverse correlation is observed between the duration spent online and the duration of sleep per day, suggesting that increased digital engagement may be associated with reduced sleep duration. A study of older adults revealed that those with digital access were associated with an average of 0.156 fewer afternoon nap hours than their counterparts in the digital divide. The association of internet access with this phenomenon was found to be nonsignificant. It has been demonstrated that older adults who have access to the internet are associated with a later sleep onset time than their counterparts who do not have access to the digital divide. Furthermore, the duration of time spent online has been shown to have a significant association with the timing of nighttime sleepiness among older adults.

**Table 2.** Associations of digital engagement with sleep schedules among older adults: results from panel regression models (brief).

Variables	Total sleep duration (h)		Afternoon nap duration (h)		Nocturnal sleep onset time^a^	
	Model 1	Model 2	Model 3	Model 4	Model 5	Model 6
Digital access, β (SE)	−0.154^b^ (0.049)	—^c^	−0.156^b^ (0.020)	—	0.664^b^ (0.076)	—
Online time^d^, β (SE)	—	−0.068^e^ (0.031)	—	0.009 (0.014)	—	0.372^b^ (0.080)
Sex, β (SE)	0.355^b^ (0.032)	0.317^b^ (0.075)	0.122^b^ (0.013)	0.035 (0.033)	−0.133^e^ (0.067)	0.159 (0.257)
At marriage, β (SE)	−0.029 (0.038)	0.245^e^ (0.111)	−0.009 (0.016)	0.102^e^ (0.051)	−0.098 (0.073)	0.021 (0.355)
Education, β (SE)	−0.022^b^ (0.004)	−0.003 (0.011)	−0.010^b^ (0.002)	−0.002 (0.005)	0.093^b^ (0.008)	0.020 (0.035)
Household, β (SE)	−0.423^b^ (0.035)	−0.318^e^ (0.131)	−0.138^b^ (0.014)	−0.087 (0.058)	0.938^b^ (0.067)	0.511 (0.396)
Age (y), β (SE)	0.005^e^ (0.002)	0.002 (0.007)	0.005^b^ (0.001)	0.003 (0.003)	−0.090^b^ (0.005)	−0.129^b^ (0.023)
Self-rated health, β (SE)	−0.008 (0.013)	−0.087^e^ (0.038)	0.015^b^ (0.006)	−0.002 (0.016)	−0.038^f^ (0.021)	0.004 (0.101)
Religious belief, β (SE)	−0.015 (0.085)	0.067 (0.217)	−0.017 (0.034)	−0.033 (0.086)	0.128 (0.132)	−0.423 (0.553)
Chronic diseases, β (SE)	−0.078^e^ (0.033)	−0.037 (0.079)	0.039^b^ (0.014)	0.089^b^ (0.034)	0.026 (0.048)	−0.063 (0.189)
Constant term, β (SE)	7.203^b^ (0.183)	7.131^b^ (0.534)	0.848^b^ (0.076)	0.675^b^ (0.229)	—	—
Year-fixed effect	Yes	Yes	Yes	Yes	—	—
Year random effect	—	—	—	—	Yes	Yes
Sample size	15,747	1306	10,183	853	15,402	1281
*R* ^2^	0.0351	0.0394	0.0531	0.0225	—	—
Wald chi-square test (*df*)	—	—	—	—	1226.42 (9)	55.17 (9)

a Nocturnal sleep onset time is an ordinal variable. Models 5 and 6 report coefficients from a random-effects ordered logit model.

b * P*<.01.

c Not applicable.

d Online time was measured as the natural logarithm of weekly hours and was only analyzed for the subsample with digital access. This explains the smaller sample sizes in models 2, 4, and 6.

e *P*<.05.

f *P*<.10.

### Analysis of the Intermediary Mechanism

To systematically examine the mechanisms through which digital access influences sleep schedules, we tested a conceptual framework that organizes five mediators into three distinct yet potentially interconnected pathways: (1) the time displacement pathway, (2) the psychosocial resource pathway, and (3) the physiological-cognitive pathway. This framework posits that digital engagement reshapes sleep by altering individuals’ daily routines, mental well-being, and physical health.

Specifically, within the time displacement pathway, we posit that digital access facilitates nonfarm payroll employment [43,44]. The time demands of such employment are hypothesized to directly compete with and reduce the time available for both nocturnal sleep and afternoon naps. Within the psychosocial resource pathway, informed by theories of aging and resource conservation, we propose that digital access enhances psychosocial well-being by reducing feelings of depression through improved social connectivity [27,28] and by maintaining functional autonomy, as measured by instrumental activities of daily living (IADLs). We hypothesize that these improvements in mental health and functional capacity may, paradoxically, lead to a reduced inclination for prolonged rest, thereby shortening sleep duration and nap time. Finally, the physiological-cognitive pathway captures the potential benefits of digital engagement on core biological and mental functions. We test whether digital access supports memory through cognitive stimulation [45,46] and improves nutrition by increasing protein intake via the convenience of e-commerce. These enhancements, while beneficial for overall health, may exert complex, and sometimes opposing, influences on sleep regulation, potentially explaining the masking effects observed in our analysis. As a prerequisite for mediation, Table S2 in Multimedia Appendix 1 confirms that digital access is significantly associated with all 5 mediators after adjusting for covariates. The following results delineate the specific indirect effects operating through these hypothesized pathways.

As shown in Table 3, the results of the mediating mechanism tests are reported herein. The results of the analysis indicated that none of the mediating effect tests for the duration of internet access on sleep schedules were significant. Therefore, the data in the table are indicative of the mediating mechanisms for the effect of digital access on the sleep schedules of older adults. The findings indicate that digital access has a significant association with the sleep patterns of older adults, evidenced by a reduction in sleep duration, afternoon nap duration, and delayed nocturnal sleep onset time. The findings suggest that the masking effect of older adults’ dietary protein intake on the attenuating effect of digital access on older adults’ sleep duration implies that digital access promotes older adults’ protein food intake, which in turn promotes sleep duration. The findings revealed a masking effect of memory on the association between digital access and sleep duration, as well as a delaying effect on sleep onset. This suggests that digital access may have a positive impact on memory in older adults. In addition, the study found a strong correlation between sleep quality and memory in older adults, supporting the hypothesis that sleep quality is a crucial factor in memory function [47,48]. Levels of depression in older adults had a masking effect on the attenuating effects of digital access on sleep duration and afternoon nap duration.

**Table 3.** Mediation analysis of the effect of digital access on sleep schedules via hypothesized mechanisms.

Sleep schedule and mediating variables		Effect	SE^a^	Percentage of effect (%)	95% CI		*P* value
Sleep duration per day							
Nonfarm payroll		−0.033	0.010	32.84	−0.052 to −0.013		<.001
Protein intake		0.009	0.004	−4.90	0.001 to 0.016		0.03
Memory		0.009	0.004	−5.76	0.001 to 0.017		0.02
Depression		0.014	0.003	−9.26	0.007 to 0.021		<.001
IADLs^b^		−0.014	0.004	9.49	−0.021 to −0.007		<.001
Afternoon nap							
Nonfarm payrolls		−0.015	0.005	8.50	−0.023 to −0.006		0.003
Depression		-0.002	0.001	1.25	−0.004 to 0.000		0.02
IADLs		−0.004	0.001	2.94	−0.007 to −0.002		<.001
Sleep onset time							
Nonfarm payrolls		0.017	0.006	6.05	0.006 to 0.029		0.02
Memory		−0.010	0.002	−2.69	−0.015 to −0.005		<.001
IADLs		0.007	0.002	1.89	0.004 to 0.010		<.001

a The significance of the indirect effects was tested using the nonparametric bootstrap method with 500 resamples. An effect is considered statistically significant if the 95% bias-corrected confidence interval (95% bootstrapped CI) does not include 0.

b IADL: instrumental activity of daily living. A higher score indicates greater difficulty, hence the negative association with digital access in the prerequisite model (Table S2 in Multimedia Appendix 1).

### Heterogeneity Analysis

Given the substantial heterogeneity in sleep behavior across sexes [49,50], and the significant heterogeneity in sleep behavior and digital access among urban and rural older adults [51-54], this study examined the associations of digital engagement with sleep schedules among older adults, with a focus on sex and urban-rural heterogeneity. As shown in Table 4, the results of the heterogeneity test were reported. It should be noted that control variables were incorporated into the model. The findings indicate that, with respect to sleep duration, digital access exerted a negligible association with the sleep patterns of female older adults. However, among male older adults, digital access demonstrated a substantial association with sleep duration. Conversely, internet access duration exhibited a significant association with female older adults, while having no discernible associations with male older adults. The associations of digital access with sleep duration were examined for 2 distinct groups of older adults: those residing in urban areas and those residing in rural areas. The findings revealed a significant association of digital access with sleep duration for the urban older adult population. However, this association was not observed among the rural older adult population. In addition, the study investigated the association between internet access duration and sleep duration. The results indicated that for rural older adults, an increase in internet access duration was associated with a decrease in sleep duration. Conversely, this association was not observed among urban older adults. In terms of sleep onset time, digital access has a more significant effect on delaying sleep onset time for older men and older adults in rural areas, while online time has a more significant effect on delaying sleep onset time for older women and older adults in urban areas, but has no significant effect on older adults in rural areas.

**Table 4.** Heterogeneity test.

Variable	Female	Male	Rural	Urban
Heterogeneity test: effects on sleep duration				
Digital access				
Coefficient	–0.067	–0.218	–0.099	–0.259
SE	0.076	0.062	0.099	0.052
*P* value	.38	<.001	.32	<.001
Sample size, n (%)	8492 (53.93)	7255 (46.07)	8679 (55.12)	7068 (44.88)
*R*^2^	0.0339	0.0344	0.0195	0.0267
Online time				
Coefficient	–0.099	–0.028	–0.175	–0.044
SE	0.045	0.043	0.088	0.033
*P* value	.03	.51	.051	.19
Sample size, n (%)	545 (41.73)	761 (58.27)	122 (9.34)	1184 (90.66)
*R*^2^	0.0648	0.0272	0.1746	0.0306
Year-fixed effect	Yes	Yes	Yes	Yes
Control variable	Yes	Yes	Yes	Yes
Heterogeneity test: effects on afternoon nap				
Digital access				
Coefficient	–0.123	–0.177	–0.148	–0.158
SE	0.031	0.025	0.039	0.022
*P* value	<.001	<.001	<.001	<.001
Sample size, n (%)	5257 (51.63)	4926 (48.37)	5555 (54.55)	4628 (45.45)
*R*^2^	0.0486	0.0545	0.0210	0.0505
Online time				
Coefficient	0.024	−0.002	0.026	0.006
SE	0.021	0.018	0.045	0.015
*P* value	.25	.93	.56	.69
Sample size, n (%)	323 (37.87)	530 (62.13)	75 (8.79)	778 (91.21)
*R*^2^	0.0726	0.0248	0.1888	0.0193
Year-fixed effect	Yes	Yes	Yes	Yes
Control variable	Yes	Yes	Yes	Yes
Heterogeneity test: effects on sleep onset time				
Digital access				
Coefficient	0.649	0.678	0.664	0.582
SE	0.110	0.107	0.128	0.101
*P* value	<.001	<.001	<.001	<.001
Sample size, n (%)	8298 (53.88)	7104 (46.12)	8498 (55.17)	6904 (44.83)
Wald chi-square test (*df*)	669.81 (9)	558.26 (9)	374.40 (9)	450.92 (9)
Online time				
Coefficient	0.519	0.258	0.258	0.386
SE	0.119	0.110	0.157	0.089
*P* value	<.001	<.001	.10	<.001
Sample size, n (%)	534 (41.69)	747 (58.31)	119 (9.29)	1162 (90.71)
Wald chi-square test (*df*)	28.93 (9)	32.58 (9)	6.17 (9)	51.40 (9)
Year-fixed effect	Yes	Yes	Yes	Yes
Control variable	Yes	Yes	Yes	Yes

## Discussion

### Principal Results

This study examined the association of digital engagement with sleep schedules among older adults, drawing upon 4 periods of CFPS data from 2014 to 2020. The findings indicated that digital engagement was associated with a reduction in sleep duration and an advancement in nocturnal sleep onset time for older adults. In addition, digital access was associated with a decrease in the length of afternoon nap duration for this demographic. The results of the heterogeneity analysis suggest the presence of internal heterogeneity in the effects of digital engagement on older adults’ sleep schedules. That is to say, the effects of digital access are not equivalent to those of hours of internet use. The association of digital access with sleep schedules among older adults was found to be more pronounced among older male adults compared to their female counterparts residing in urban areas. Similarly, the impact of internet use hours on sleep schedules was found to be more significant for older female adults compared to their male counterparts residing in urban areas. These findings suggest that digital engagement may act as a “double-edged sword” for older adults’ sleep: while digital access is associated with more balanced sleep schedules, excessive internet use may undermine sleep duration and delay sleep onset.

### Limitations

Despite the fact that the tracking data facilitated causal inference, this study was not without its limitations. First, and most importantly, the observational nature of our data prevents definitive causal conclusions. Although we used panel FE models and a random-effects ordered logit model to control for time-invariant confounders, reverse causality remains a plausible alternative explanation. It is possible that older adults who experience insomnia or have poorer sleep quality may turn to digital devices during wakeful periods, which could partly explain the observed associations between digital engagement and delayed or shorter sleep.

Second, due to the nature of the CFPS as a comprehensive household survey, rather than a survey specifically targeting older adults, the data are not well-targeted, and the sample size for the digitally engaged subgroup is relatively small. Third, for the same reasons as the previous point, the clinical significance of this study is relatively poor; for example, the study’s limitations include its failure to consider the physiological mechanisms through which digital engagement affects older adults’ sleep schedules. Finally, the lack of a new database from the CFPS for years beyond 2020, despite the study’s completion, has hindered the identification of potentially evolving effects of digital engagement. Despite having completed the survey, the timeliness of this study is less than ideal, as evidenced by the year in which it was conducted: 2020.

The generalizability of our findings may be influenced by the relatively low rate of digital access (13.5%) in our analytical sample, which is an average across the survey waves from 2014 to 2020. This rate is substantially lower than the figures reported in contemporaneous Western populations [4,5]. It is, however, representative of the digital inclusion landscape for older adults in China during this specific period, reflecting a historically lower but rapidly growing penetration rate. Consequently, our findings are most directly generalizable to contexts with similar levels of digital adoption among older adults. As global digital engagement continues to rise, the associations observed here may evolve.

### Comparison With Prior Work

A body of research has examined the impact of digital engagement on sleep duration in older adults [55]. However, the existing literature is not sufficiently comprehensive to determine the influence of digital engagement on the overall sleep behavior of this demographic. A number of studies have examined the efficacy of automated, web-based insomnia intervention programs in promoting sleep in older adults [56]. However, these studies have not directly addressed the impact of digital engagement on sleep quality in this demographic. A paucity of research has been conducted on the direct impact of digital engagement on the sleep patterns of older adults.

The mediation analysis unveils the complex mechanisms underpinning this “double-edged sword” effect, revealing competing pathways through which digital access influences sleep. On one hand, digital engagement appears to erode sleep time through time-displacement and activity-enabling pathways. Specifically, it facilitates nonfarm employment and maintains functional capacity (as measured by IADLs), both of which compete with time available for rest, leading to shorter nocturnal sleep, reduced napping, and later bedtimes. On the other hand, digital access also seems to promote psychosocial and physiological resources that are generally beneficial for health, yet these exert countervailing (masking) effects on sleep. For instance, it is associated with improved memory, higher protein intake, and reduced depressive symptoms—factors that are themselves positively correlated with better sleep quality or duration. The net negative association observed in our main results suggests that the sleep-disrupting effects of time displacement and heightened activity may outweigh the protective effects conferred by these enhanced resources in the current cohort of older adults.

In light of the mounting popularity of digital devices and the expanding scope of digital engagement among older adults, it is imperative to examine the impact of digital engagement on sleep quality in this demographic. This research not only addresses a significant practical concern but also contributes to the theoretical underpinnings of related research domains. Furthermore, our mediation analysis provides empirical evidence for the potential mechanisms underpinning this “double-edged sword” effect, which can be interpreted through several theoretical lenses. The finding that digital access reduces sleep duration through increased engagement in nonfarm employment aligns with theories of time displacement, where digital connectivity fosters economic opportunities that directly compete with time traditionally allocated for rest [57]. Conversely, the observed masking effects of improved protein intake, memory, and reduced depressive mood suggest a more complex relationship. This can be framed within a social-ecological model: digital access appears to enhance psychosocial and physiological resources at the individual level [58], which are generally protective for health, yet simultaneously introduces competing time demands and cognitive stimulation at the behavioral level that disrupt sleep patterns. The net negative effect on sleep duration suggests that the time-displacing and cognitively arousing effects of digital engagement may outweigh its potential benefits for sleep in this population. Finally, the mechanism via IADL improvement—whereby better functional capacity leads to reduced sleep—resonates with the activity theory of aging, proposing that greater engagement in life activities can reduce the time and need for sleep [59]. These competing mechanisms highlight the nuanced role of digital technology in older adults’ lives, acting simultaneously as a tool for resource enhancement and a source of behavioral disruption.

In this study, we operationalized digital engagement as the length of digital access and internet use, and we defined older adults’ sleep as the sleep schedule, that is, the length of daily sleep, the length of afternoon nap duration, and the time of falling asleep at night. This study systematically and comprehensively focuses on the impact of digital engagement on the sleep schedule of older adults, which meets the needs of the real world and fills in the shortcomings of related research.

### Implications and Contribution

The findings of the mediating mechanism test suggest that digital access has a significant impact on the total sleep duration of older adults. It does so by promoting nonfarm employment and reducing their IADLs. However, this effect is obscured by factors such as depression levels, protein intake, and memory levels. Furthermore, digital access has been observed to curtail the naptime of older adults by fostering nonfarm employment, mitigating depression, and reducing IADLs. In addition, it has been demonstrated that digital access delays nocturnal sleep onset time in older adults by promoting nonfarm employment and reducing IADLs. This effect is, however, masked by depression levels in older adults.

The findings of the mediating effect unveil two issues that have not been the focal point of prior research. First, nonfarm employment resulting from digital access, while facilitating older adults’ access to economic resources, is detrimental to their overall achievement of healthier sleep schedules. Second, digital access promotes older adults’ physiological well-being, whereas superior physiological well-being permits older adults to engage in more activities that may instead result in shorter sleep duration and falling asleep too late.

Subgroup characterization of sample characteristics suggests that digital access may contribute to a more balanced sleep duration, nap duration, and healthier sleep onset time for older adults overall, but also contribute to later sleep onset time and less sleep duration than older adults across the digital divide. Studies have shown that appropriate afternoon nap duration length is beneficial to cognitive health in older adults [60,61], and appropriate daily sleep length is beneficial to physical and mental health in older adults [62-64], while going to bed too late increases health risks in older adults [65], so it is possible to identify heterogeneity in the impact of digital access and internet use on older adults’ sleep schedules, and the different health implications: digital access has been shown to promote healthier sleep schedules among older adults. However, excessive internet use can have adverse health consequences. While digital access contributes to healthier sleep schedules, excessive internet use can hinder the achievement of a healthy sleep schedule among older adults, leading to digital addiction.

The results of the heterogeneity analysis demonstrate the impact of sex and urban-rural inequalities in digital access. Disadvantaged groups, in their efforts to access digital resources, require greater social resources. This, in turn, could lead to these groups being more likely to be overly digitally engaged, which, in turn, leads to less healthy overall sleep schedules.

### Conclusions

In this study, digital engagement was associated with shorter sleep duration and afternoon nap duration, as well as later sleep onset time in older adults. However, the associations between digital engagement and older adults’ sleep schedules exhibit internal heterogeneity, with digital access favoring healthier sleep schedules and excessive hours of internet use potentially resulting in less favorable outcomes.

In summary, it is imperative for policymakers to acknowledge the dual impact of digital engagement on the sleep patterns of older adults. On the one hand, promoting digital penetration, which is associated with more balanced sleep schedules in some contexts, remains important. On the other hand, there is a need to popularize sleep hygiene and health habits among older adults who have already achieved digital penetration through digital media.

## Supplementary material

10.2196/79731 Multimedia Appendix 1 Baseline characteristics, regression models, mediation, and heterogeneity analyses examining the associations between digital engagement and sleep patterns in older adults.

10.2196/79731 Checklist 1 STROBE checklist.

## Abbreviations

CFPS: China Family Panel Studies
FE: fixed effects
IADL: instrumental activity of daily living
