# Supplementary material for: The Double-Edged Sword of Digital Engagement—How Digital Access and Internet Use Reshape Sleep Schedules and Underlying Mechanisms in Older Adults: Longitudinal Observational Study
Source: JMIR Aging. 2025 Nov 5;8:e79731. doi: 10.2196/79731 (PMC12588592; doi:10.2196/79731)
Supplement: Multimedia Appendix 1 [file aging-v8-e79731-s001.docx]

**Tabel S1.** Associations of Digital Engagement with Sleep Schedules Among Older Adults: Results from Panel Regression Models (Detailed)

| Variables | Sleep duration | | | | Afternoon nap | | | | Sleep onset time | | | |
| --- | --- | --- | --- | --- | --- | --- | --- | --- | --- | --- | --- | --- |
|  | (1) | p-value | (2) | p-value | (3) | p-value | (4) | p-value | (5) | p-value | (6) | p-value |
| Digital access | -0.154*** | .002 |  |  | -0.156*** | <.001 |  |  | 0.664*** | <.001 |  |  |
|  | (0.049) |  |  |  | (0.020) |  |  |  | (0.076) |  |  |  |
| Online time |  |  | -0.068** | .027 |  |  | 0.009 | .53 |  |  | 0.372*** | <.001 |
|  |  |  | (0.031) |  |  |  | (0.014) |  |  |  | (0.080) |  |
| Gender | 0.355*** | <.001 | 0.317*** | <.001 | 0.122*** | <.001 | 0.035 | .30 | -0.133** | .046 | 0.159 | .54 |
|  | (0.032) |  | (0.075) |  | (0.013) |  | (0.033) |  | (0.067) |  | (0.257) |  |
| At marriage | -0.029 | .438 | 0.245** | .028 | -0.009 | .569 | 0.102** | .047 | -0.098 | .18 | 0.021 | .95 |
|  | (0.038) |  | (0.111) |  | (0.016) |  | (0.051) |  | (0.073) |  | (0.355) |  |
| Education | -0.022*** | <.001 | -0.003 | .761 | -0.010*** | <.001 | -0.002 | .68 | 0.093*** | <.001 | 0.020 | .58 |
|  | (0.004) |  | (0.011) |  | (0.002) |  | (0.005) |  | (0.008) |  | (0.035) |  |
| Household | -0.423*** | <.001 | -0.318** | .015 | -0.138*** | <.001 | -0.087 | .13 | 0.938*** | <.001 | 0.511 | .20 |
|  | (0.035) |  | (0.131) |  | (0.014) |  | (0.058) |  | (0.067) |  | (0.396) |  |
| Age | 0.005** | .022 | 0.002 | .769 | 0.005*** | <.001 | 0.003 | .41 | -0.090*** | <.001 | -0.129*** | <.001 |
|  | (0.002) |  | (0.007) |  | (0.001) |  | (0.003) |  | (0.005) |  | (0.023) |  |
| Self-rated health | -0.008 | .564 | -0.087** | .021 | 0.015*** | .007 | -0.002 | .92 | -0.038* | .07 | 0.004 | .97 |
|  | (0.013) |  | (0.038) |  | (0.006) |  | (0.016) |  | (0.021) |  | (0.101) |  |
| Religious belief | -0.015 | .857 | 0.067 | .759 | -0.017 | .613 | -0.033 | .70 | 0.128 | .33 | -0.423 | .44 |
|  | (0.085) |  | (0.217) |  | (0.034) |  | (0.086) |  | (0.132) |  | (0.553) |  |
| Chronic diseases | -0.078** | .018 | -0.037 | .641 | 0.039*** | .004 | 0.089*** | .01 | 0.026 | .59 | -0.063 | .74 |
|  | (0.033) |  | (0.079) |  | (0.014) |  | (0.034) |  | (0.048) |  | (0.189) |  |
| cons | 7.203*** | <.001 | 7.131*** | <.001 | 0.848*** | <.001 | 0.675*** | .003 |  |  |  |  |
|  | (0.183) |  | (0.534) |  | (0.076) |  | (0.229) |  |  |  |  |  |
| Year fixed effect | Y |  | Y |  | Y |  | Y |  |  |  |  |  |
| Year random effect |  |  |  |  |  |  |  |  | Y |  | Y |  |
| Sample size | 15747 |  | 1306 |  | 10183 |  | 853 |  | 15402 |  | 1281 |  |
| R^2^ | 0.0351 |  | 0.0394 |  | 0.0531 |  | 0.0225 |  |  |  |  |  |
| Wald chi2 |  |  |  |  |  |  |  |  | 1226.42 |  | 55.17 |  |

**Tabel S2.** The correlation between digital access and mediating variables^a^

| Mediator Variables | Digital Access Model (coef/SE/p-value) |
| --- | --- |
| Non-farm payroll | 0.120/0.018/<.001 |
| Protein intake | 0.047/0.016/<.01 |
| Memory | 0.335/0.035/<.001 |
| Depression | -0.659/0.139/<.001 |
| IADL | -0.139/0.039/<.001 |

^a^The control variables and the fixed effect of the year were controlled.
